# Detectors assessment for stereotactic radiosurgery with cones

**DOI:** 10.1002/acm2.12449

**Published:** 2018-09-14

**Authors:** Nicolas Garnier, Régis Amblard, Rémy Villeneuve, Rodolphe Haykal, Cécile Ortholan, Philippe Colin, Anaïs Gérard, Sarah Belhomme, Franck Mady, Mourad Benabdesselam, Benjamin Serrano

**Affiliations:** ^1^ Medical Physics Department Princess Grace Hospital Center Monaco Monaco; ^2^ Institut de Physique de Nice Côte d'Azur University Parc Valrose Nice France; ^3^ Radiotherapy Department Princess Grace Hospital Center Monaco Monaco; ^4^ Medical Physics Department Centre Antoine Lacassagne Nice France; ^5^ Medical Physics Department Institut Bergonié Bordeaux France

**Keywords:** beam profiles, detectors, Monte Carlo, output factor, percent depth dose, stereotactic cone

## Abstract

The purpose of this work is to assess eight detectors performance for output factor (OF), percent depth dose (PDD), and beam profiles in a 6‐MV Clinac stereotactic radiosurgery mode for cone irradiation using Monte Carlo simulation as reference. Cones with diameters comprised between 30 and 4 mm have been studied. The evaluated detectors were ionization chambers: pinpoint and pinpoint 3D, diodes: SRS, P and E, Edge, MicroDiamond and EBT3 radiochromic films. The results showed that pinpoints underestimate OF up to −2.3% for cone diameters ≥10 mm and down to −12% for smaller cones. Both nonshielded (SRS and E) and shielded diodes (P and Edge) overestimate the OF respectively up to 3.3% and 5.2% for cone diameters ≥10 mm and in both cases more than 7% for smaller cones. MicroDiamond slightly overestimates the OF, 3.7% for all the cones and EBT3 film is the closest to Monte Carlo with maximum difference of ±1% whatever the cone size is. For the profiles and the PDD, particularly for the small cones, the size of the detector predominates. All diodes and EBT3 agree with the simulation within ±0.2 mm for beam profiles determination. For PDD curve all the active detectors response agree with simulation up to 1% for all the cones. EBT3 is the more accurate detector for beam profiles and OF determinations of stereotactic cones but it is restrictive to use. Due to respectively inappropriate size of the sensitive volume and composition, pinpoints and diodes do not seem appropriate without OF corrective factors below 10 mm diameter cone. MicroDiamond appears to be the best detector for OF determination regardless all cones. For off‐axis measurements, the size of the detector predominates and for PDD all detectors give promising results.

## 1 INTRODUCTION

Effectiveness of Linac‐based stereotactic radiosurgery (SRS) with small cone sizes (few millimeters) brings to more and more frequent use, especially for brain treatments (metastases, trigeminal neuralgia, arteriovenous malformation (AVM), and other brain localizations).^1^, ^2^, ^3^ To ensure the quality of these treatments with small field sizes, measurements of percentage depth dose (PDD) curves, tissue–phantom ratios, profiles, and output factors (OF) should be well achieved in spite of the size and composition of the detectors.^4^, ^5^, ^6^, ^7^ In this study we will focus on some high dosimetry accuracy measurements of OF, PDD, and off‐axis measurements for use of small photon fields in SRS cone irradiation with diameters between 30 and 4 mm.

The required determination of OF will not target on correction factors of the OF as mentioned in several research groups.^4^, ^8^ Our purpose is to assess the variation in performance of eight detectors for OF, but also to study for some of these detectors their performance for PDD and beam profiles measurements in a clinical 6‐MV linear accelerator photon beam using the PENELOPE Monte Carlo (MC) code^9^ as reference.

## 2 MATERIALS AND METHODS

### 2.A Conventional Linac‐based device

Measurements were performed by means of a linear accelerator Clinac 2100C (Varian Medical System, Pal Alto, CA) at 6‐MV photon beam with an energy index (TPR_20,10_) of 0.669. The Linac is equipped with an accessory slot mounted cone system developed by BrainLAB. The cone set consists of ten cones with diameters of 30, 25, 20, 17.5, 15, 12.5, 10, 7.5, 5, and 4 mm at the isocenter. The field size defined by the jaws behind the cones was set to 4 × 4 cm square. The Linac nominal dose rate was fixed at 600 MU/min.

### 2.B List of used detectors

Seven active detectors and a passive one (Radiochromic film EBT3) were used (Table 1).

**Table 1:** Summary of detectors characteristics

Label	Type	Active volume dimensions	Effective point	Material	Zeff
PinPoint	Air filled‐ionization chamber	Ø 2 mm	On detector axis, 3.4 mm from chamber tip	Wall: 0.57 mm PMMA	7.64
31014		5 mm height		0.09 mm graphite	
				Electrode: Ø 0.3 mm Al	
PTW		15 mm^3^			
PinPoint 3D	Air‐filled ionization chamber	Ø 2.9 mm	On detector axis, 2.4 mm from chamber tip	Wall: 0.57 mm PMMA	7.64
31016		2.9 mm height		0.09 mm graphite	
PTW		16 mm^3^		Electrode: Ø 0.3 mm Al	
Diode SRS	Unshielded diode (USD)	Disk, Ø 1.13 mm	On detector axis, 1.31 mm from detector tip	Silicon	14
60018					
		250 μm thick			
PTW		0,3 mm^3^			
Diode P	Shielded diode (SD)	Disk, Ø 1.13 mm	On detector axis, 2 mm from detector tip	Silicon	14
60008					
		2.5 μm thick			
		0,0025 mm^3^			
PTW					
Diode E	Unshielded diode (USD)	Disk, Ø 1.13 mm	On detector axis, 1.33 mm from detector tip	Silicon	14
60017					
		30 μm thick			
PTW		0,03 mm^3^			
Diode Edge Sun Nuclear	Shielded diode (SD)	Square, 0.8 × 0.8 mm²	On detector axis, 0.2 mm from detector tip	Silicon	14
		30 μm thick			
		0,019 mm^3^			
MicroDiamond	Synthetic diamond	Disk, Ø 2.2 mm	On detector axis, 1 mm from detector tip	Diamond	6
60019					
		1 μm thick			
PTW		0,004 mm^3^			
EBT3 film GAFCHROMIC Ashland	Radiochromic film	278 μm thick	Center of the film	H(56.8), Li(0.6), C(27.6), O(13.3), Al (1.6) (% of each atom)	7.26

(water: Zeff = 7.42).

Diodes and MicroDiamond detectors were used in axial orientation while the ionization chambers were used in both axial and radial positions.

### 2.C Setup and measurements

OF, PDD, and profiles measurements were performed with a 90‐cm source–surface distance. OF and profiles were achieved at 10 cm depth in water.

#### 2.C.1 Active detectors

Measurements were made using a PTW MP3 scanning water phantom controlled by Mephysto software. “True Fix” system was used to position detectors. This system allows accurate positioning of effective points of measurements of various detectors on the surface of water phantoms.

#### 2.C.2 Passive detector

Measurements with GAFCHROMIC EBT3 were positioned perpendicular to the beam axis in a solid water equivalent phantom of 30 × 30 × 30 cm. Films were cut into squares of 5 cm on each side, 24 h before irradiation. The upper right corner was marked at the time the film was cut to define its orientation. Since the relation between pixel value and absorbed dose is non‐linear, a calibration dose is necessary. The calibration of EBT3 films is performed by doing eight expositions to a 6‐MV beam for a dose range [0–500 cGy]. For stabilization of the films response, 48 h standby times were observed. Films for calibration and analyze were scanned with Epson 11000XL flatbed scanner in transmission mode with a resolution of 150 dpi and 48‐bit RGB format. Exploitation of the films was performed with Film QA Pro software (Ashland) including multichannel correction.^10^ The red color channel was used to calculate the absorbed dose on EBT3 films.

### 2.D OF measurements

In this work, the output factor (OF_coll_) was defined by Eq. (1):

(1) $$OF_{\mathrm{coll}}^{\det}=\frac{D_{\mathrm{coll}}^{\det}}{D_{30mm}^{\det}}$$

where *D*_*coll*_ represents the measured dose by the detector (*det*) for each collimator and *D*_*30mm*_ the reference dose measured with the 30‐mm diameter collimator. The latter was used as a reference instead of the standard 10 × 10 cm field for two reasons: (a) it is closer to the small fields while there is still sufficient electronic equilibrium and good agreement between measurements made with various types of detectors,^11^ (b) in this way, it is not necessary to take into account the contribution of backscatter from X‐Y jaws to the beam monitor in the Monte Carlo simulation because jaws position was fix whatever the cone diameter studied.

#### 2.D.1 Active detectors

After each detector or cone changes, in‐plane and cross‐plane, profiles were done to center the detector. By means of PTW UNIDOS Webline electrometer for all active detectors, dose measurements were achieved with 100 MU and averaged over a series of at least three repeated runs on different days.

#### 2.D.2 Passive detector

In order to find the beam center of the film and to place automatically a region of interest (ROI), a computer code was written on MATLAB software. The code search along the in‐plane and cross plane directions was to find out the beam center from the full width at half‐maximum (FWHM) of the profiles. The ROI size was 0.6 mm for the 4 mm cone diameter and 1 mm for all the others. The film absolute dose was evaluated by taking the average of the voxels dose on the ROI. Films measurements were averaged over a series of thirteen irradiation times on different days.

Monitor units (MU) number issued for each cone was calculated so as to obtain an absorbed dose in the film of about 4 Gy whatever the cone size is (Table 2). MU number was determined from diode SRS results.

**Table 2:** The number of monitor units delivered according to the cone diameter

Cone diameter (mm)	30	25	20	17.5	15	12.5	10	7.5	5	4
MU number	576	586	599	607	619	639	668	721	830	925

This method allows to work in the ideal dose range for the film and to obtain the same signal to noise ratio and thus the same uncertainty whatever the cone size is. For films, the output factor (OF_coll_) was defined by Eq. (2):

(2) $$OF_{\mathrm{coll}}^{EBT3}=\frac{D_{\mathrm{coll}}^{EBT3}}{D_{30mm}^{EBT3}}\times \frac{MU_{30mm}}{MU_{\mathrm{coll}}}$$

Where *D*_*coll*_ represents the EBT3 measured dose for a given collimator, D_30mm_ corresponds to the EBT3 dose reference measured with the 30‐mm collimator. *MU*_*30mm*_ is the MU number used with the 30‐mm collimator and *MU*_*coll*_ the one corresponding to the studied collimator.

### 2.E PDD measurements

PDD measurements were performed with a water phantom. Precautions should be taken for the PDD measurements as explained by Khelashvili et al.^12^ on the gantry tilt. Considering this, several beam profiles (for in‐plane and cross‐plane positions) were made at different depths (2, 10, and 30 cm) so as to ensure the best alignment. All active detectors were positioned to ensure that the nominal depth corresponded to the effective detector's point of measurement. For this measurement, the film was not used because it is not suitable due to the irradiated film length. Indeed, the inhomogeneity of the scanner response on this length is not acceptable.

### 2.F Beam profiles measurements

Profiles measurements were achieved with a water phantom for active detectors and a solid water equivalent one for the films.

To analyze the film profiles, we developed a routine which detects the circular field center and makes 18 coaxial profiles, passing through the center, spaced by 10 degrees angle. This method makes a reduction of the statistical noise without creating several parallel profiles and thus increasing the “sensitive volume” of the detector.

### 2.G Monte Carlo simulation

The PENELOPE code^9^ is one of the several general‐purpose MC packages available intended for simulation of particle transport in radiation therapy. This code is reliable mostly due to the advanced physics and algorithms for their electron transport component. Here, the user‐code PenEasy^13^ was used. PenEasy is a modular, general‐purpose main program for the PENELOPE Monte Carlo system including various source models, tallies and variance‐reduction techniques (VRT). The code includes a new geometry model for performing quadratic and voxelized geometries.

**Figure 1:**
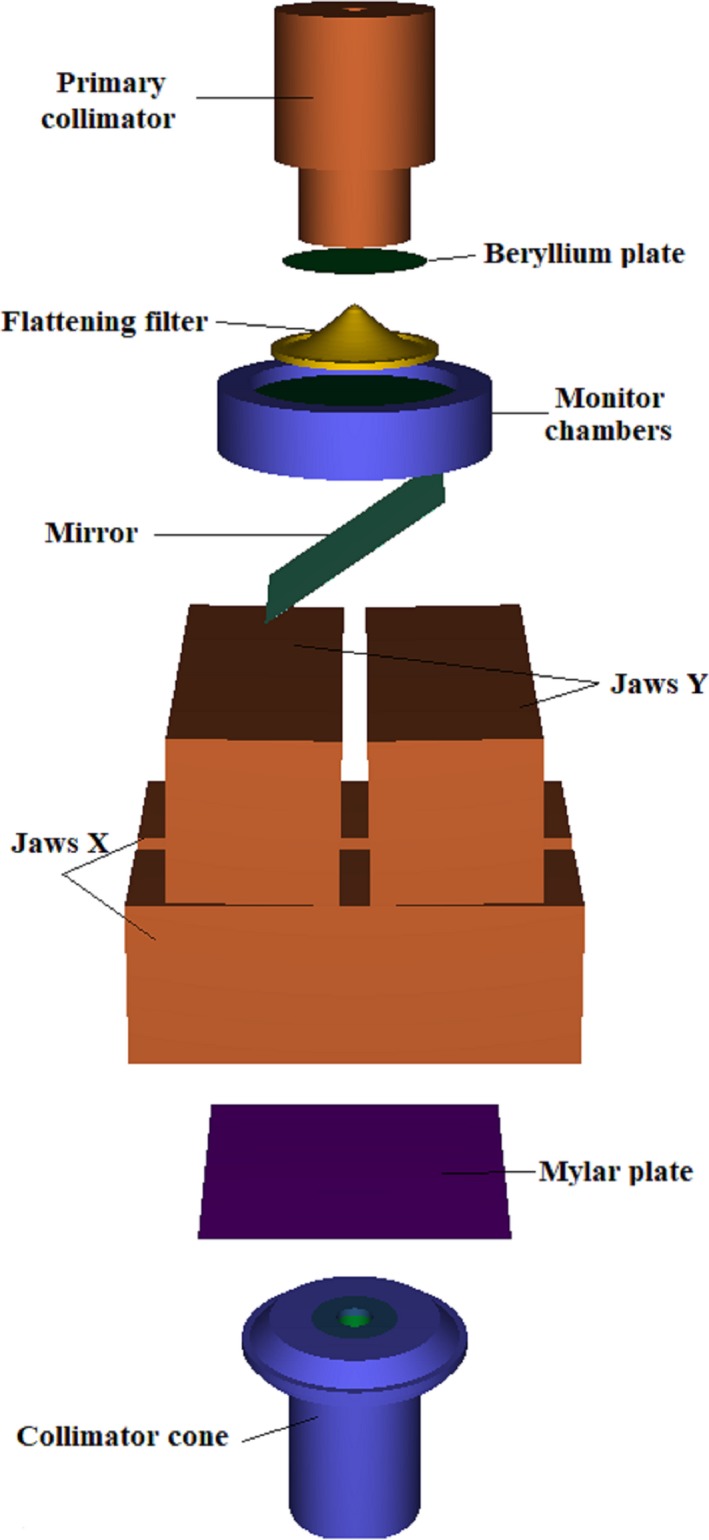
A three‐dimensional view of the Varian Clinac 2100C geometry operating in photon mode with a cone.

The treatment heads of the Clinac 2100C were simulated according to manufacturer specifications. The geometry of the accelerator is composed of: target, primary collimator, beryllium plate, flattening filter, monitor chambers, mirror, jaws, Mylar plate, and collimator cone (see Fig. 1).

Source characteristics of the 6‐MV photon beam were determined iteratively by varying the energy of the primary electron beam, its energetic dispersion, and its shape.^14^, ^15^, ^16^, ^17^ Parameters used for the primary electron beam was based on a monoenergetic 5.95 MeV beam impinging on the target with a Gaussian spatial distribution and a FWHM of 1 mm. With these parameters, PDD and profile comparison between simulation and measurement do not exceed ±1% in homogenous water phantom for field sizes comprised between 2 × 2 cm and 20 × 20 cm.

Interaction forcing variance reduction and phase‐space file (PSF) techniques were used in the simulation of the treatment head. Bremsstrahlung event is forced in target with a factor of 20. It means that the interaction probability of this event will be increased by a factor of 20. The phase space was realized just before collimator cone because the geometry is not modified upstream. PSF is read several times (between two and five times) in order to obtain the desired statistical uncertainty. All the variance reduction techniques applied were tested in order to prove that they do not change the physics of the calculation and they provide an unbiased estimate of any scored quantity.

The transport energy cutoff of photons and charged particles were respectively 10 and 100 keV. The threshold energies for charged radiative particle and inelastic collisions were set equal to 10 keV. The parameters C1 and C2, modulating the limit between detailed and condensed charged particle simulation, were set to 0.05.

The small volumes of water used for the calculation of *D*_*coll*_ in Eq. (1) were taken to be a cube with 1 mm side centered in the beam axis.

## 3 RESULTS

### 3.A Statistical and reproducibility aspects of the OF, PDD and beam profiles determination

For the OF, PDD, and profiles determination with Monte Carlo simulations, the statistical uncertainties (type‐A) were lower than 0.8%.

In the case of active detectors (diodes, ionization chambers, and MicroDiamond), all the measurements were repeated three times in a water tanker at three different days. The uncertainty based on the TRS‐398 report uncertainties^18^ were respectively 0.1% for the pinpoint chambers, 0.2% for the diodes (SRS, P, E, and Edge), and less than 0.3% for the MicroDiamond.

EBT3 radiochromic film measurements for OF estimation were averaged over a series of 13 irradiations. This passive detector is known to have noise uncertainty^10^, ^19^ but in accordance with film dosimetry multichannel correction,^10^, ^20^, ^21^ we obtained a relative uncertainty less than 1.5%.

### 3.B OF results

Table 3 presents the results of the OF measurements for cone diameters ranging between 30 and 4 mm, performed with the passive detector, the active ones, and the Monte Carlo simulations on a Clinac 2100C linear accelerator.

**Table 3:** OF simulated and measured with all detectors for different cone size diameters

Detector	Cone diameter (mm)									
	30	25	20	17.5	15	12.5	10	7.5	5	4
PinPoint (axial)	1.000	0.982	0.958	0.941	0.915	0.875	0.824	0.741	0.605	0.512
PinPoint (radial)	1.000	0.982	0.954	0.933	0.905	0.860	0.801	0.699	0.509	0.379
PinPoint 3D (axial)	1.000	0.982	0.956	0.938	0.910	0.870	0.816	0.727	0.580	0.474
PinPoint3D(radial)	1.000	0.985	0.955	0.933	0.903	0.854	0.793	0.693	0.529	0.415
Diode SRS	1.000	0.983	0.963	0.949	0.931	0.901	0.863	0.799	0.694	0.623
Diode P	1.000	0.984	0.967	0.955	0.940	0.914	0.878	0.822	0.710	0.615
Diode E	1.000	0.983	0.963	0.950	0.930	0.900	0.863	0.798	0.694	0.626
Edge	1.000	0.985	0.965	0.954	0.936	0.910	0.872	0.808	0.698	0.622
MicroDiamond	1.000	0.983	0.962	0.948	0.928	0.896	0.855	0.788	0.672	0.597
EBT3	1.000	0.986	0.961	0.945	0.924	0.884	0.838	0.758	0.652	0.579
MC simulation	1.000	0.983	0.959	0.944	0.920	0.885	0.835	0.764	0.648	0.581

**Figure 2:**
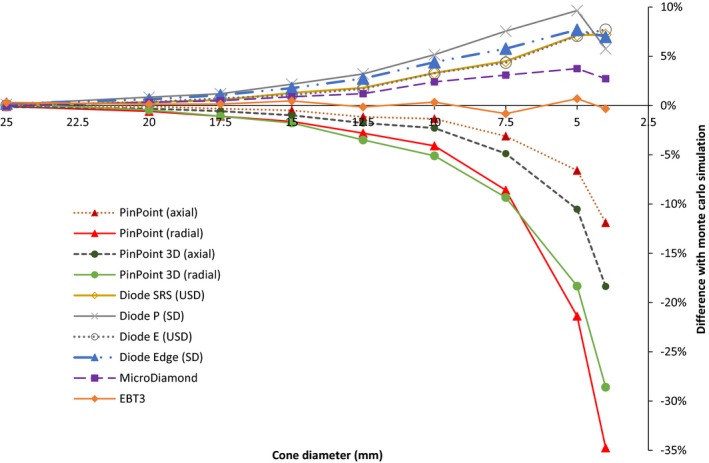
Comparison of all the OF measured for the passive and active detectors with the gold standard Monte Carlo simulation OF. Measurements with the pinpoint and pinpoint 3D detectors were done by positioning them parallel and perpendicular to the photon beam axis.

Figure 2 shows the comparison between the OF measured by all the detectors and the OF simulated by Monte Carlo considered as the reference.

One can observe large variations: −35% to +10% (Fig. 2) as described in many publications.^4^, ^5^, ^6^, ^7^, ^8^

#### 3.B.1 Ionization chambers: pinpoint and pinpoint 3D

We used two different orientations of the pinpoint chambers, perpendicular (radial) and parallel (axial) to the irradiation beam. The pinpoint and the pinpoint 3D detectors are quite similar in their characteristics with a very similar sensitive volume. However, pinpoint 3D geometry was developed so as to get a better isotropic response. Indeed, Fig. 2 shows better results of the OF measurements in parallel (axial) position for both types of pinpoint detectors. The difference between the two positions of pinpoint detectors in the OF measurements are, for cone diameters less than 10 mm : 3% (10 mm cone) up to 23% (4 mm cone). For the pinpoint 3D detector this difference is, 2.8% (10 mm cone) up to 10% (4 mm cone). One can observe a smaller response gap for the pinpoint 3D.

Oncomparing the response of the chambers to MC simulations, the detectors in radial position, show an underestimation of the OF up to −2.3% for cone diameters ≥10 mm and down to −12% for smaller ones in the case of the pinpoint.

#### 3.B.2 Diodes: E, P, SRS, and Edge

Compared to MC simulation, the nonshielded diodes (SRS and E) as well as the shielded ones (P and Edge) overestimate OF measurements by respectively up to 3.3% and 5.2% for cone diameters ≥10 mm. For smaller cones OF exceeds 7% in whatever diode used.

As one can observe in Fig. 2, the shielded diodes further overestimate the OF than the nonshielded ones. The shielded component known as interesting for large fields (>10 × 10 cm) is rather a handicap for studying with small cones.^22^

#### 3.B.3 MicroDiamond

The output factor measured with the MicroDiamond detector slightly overestimates the value, in comparison with the MC simulation, up to 3.7% for all the cones (Fig. 2). This overresponse is also observed by Ralston et al.^5^ For the smallest cones (4 and 5 mm diameters), this active detector presents promising results.

#### 3.B.4 Radiochromic EBT3 film

Using radiochromic EBT3 film to determine OF is commonly accepted and validated in the literature.^6^, ^8^, ^23^ Then, as expected, the OF measurement with this film, are the closest to MC simulations than those obtained with the active detectors. The maximum difference with MC is ±1% whatever the cone size is.

### 3.C Beam profiles results

**Figure 3:**
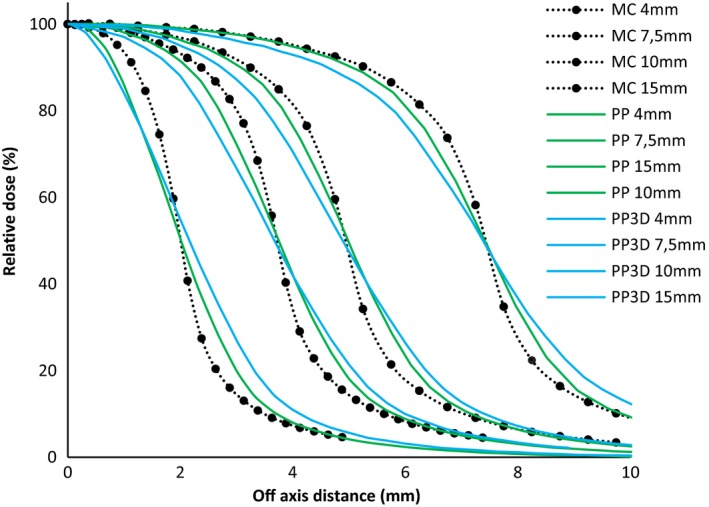
Beam profiles comparison between the two ionization chambers (pinpoint and pinpoint 3D) and Monte Carlo simulation for cone diameters of 4, 7.5, 10, and 15 mm.

Tables 4 show respectively the penumbral widths (distance between the 80% and 20% points) of measured beam profiles using all the detectors and simulated with Monte Carlo for all the different cone size diameters on a Clinac 2100C linear accelerator. One can see (Table 4 and Fig. 3) that the pinpoint and pinpoint 3D detectors overestimate the penumbra (up to more than 1 mm for the pinpoint 3D) due of their relatively large size. For all the other detectors, within the experimental uncertainties, we obtain a good agreement with MC simulations (less than 0.45 mm). In the case of the EBT3 film, the penumbra difference with MC is even less than 0.1 mm.

**Table 4:** Penumbral widths simulated and calculated with all detectors for different cone size diameters

	Penumbra widths (mm) 80% to 20%									
	Cone diameter (mm)									
	4	5	7.5	10	12.5	15	17.5	20	25	30
Monte Carlo	1.16	1.28	1.55	1.82	1.91	2.11	2.16	2.23	2.3	2.37
EBT3	1.24	1.31	1.6	1.91	1.98	2.11	2.19	2.27	2.32	2.4
Diode E	1.2	1.29	1.48	1.62	1.83	1.86	1.86	1.95	1.94	2.05
Diode P	1.34	1.5	1.68	1.81	1.95	2.05	2.07	2.07	2.15	2.15
Diode SRS	1.27	1.36	1.54	1.66	1.81	1.92	1.95	2.02	2.05	2.13
Diode Edge	1.23	1.33	1.53	1.77	2.06	1.97	1.96	1.98	2.03	2.13
PP (radial)	1.82	1.95	2.2	2.35	2.49	2.66	2.77	2.77	2.91	3.07
PP3D (radial)	2.17	2.36	2.67	2.86	3.03	3.26	3.43	3.35	3.44	3.47
MicroDiamond	1.61	1.69	1.84	1.97	2.12	2.2	2.25	2.26	2.37	2.41

In Table 5, the FWHM for each cone diameter is calculated using the measured beam profiles of each detector. The difference between the FWHM values for all the detectors and that of MC are within ±0.4 mm. Note that in the case of the diodes (P, E, SRS, and Edge), this difference in FWHM values is ±0.2 mm.

**Table 5:** FWHM of profiles simulated and calculated with all detectors for different cone size diameters

	FWHM (mm)									
	Cone diameter (mm)									
	4	5	7.5	10	12.5	15	17.5	20	25	30
Monte Carlo	4.02	4.99	7.44	9.88	12.33	14.85	17.32	19.88	24.89	29.93
EBT3	3.94	4.91	7.25	9.79	12.19	14.74	17.31	19.76	24.52	29.7
Diode E	3.97	4.93	7.46	9.89	12.16	14.86	17.38	19.84	24.71	29.89
Diode P	3.9	5	7.37	9.87	12.18	14.83	17.47	19.87	24.71	29.94
Diode SRS	3.82	4.9	7.47	9.98	12.22	14.9	17.43	19.93	24.73	29.91
Diode Edge	3.88	4.93	7.41	9.87	12.12	14.82	17.36	19.85	24.7	29.92
PP (radial)	4.03	4.98	7.5	9.97	12.2	14.82	17.35	19.81	24.65	29.9
PP3D (radial)	4.33	5.12	7.35	9.78	12.03	14.76	17.2	19.6	24.47	29.75
MicroDiamond	3.78	4.9	7.4	9.92	12.18	14.84	17.39	19.87	24.7	29.92

**Figure 4:**
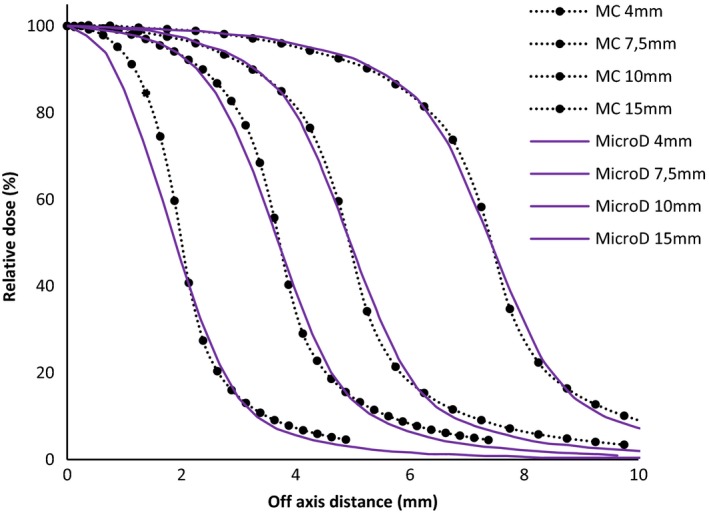
Beam profiles comparison between MicroDiamond and Monte Carlo simulation for cone diameters of 4, 7.5, 10, and 15 mm.

Figures 3 and 4 show the beam profiles comparison between the two ionization chambers (pinpoint and pinpoint 3D) respectively, and the MicroDiamond with Monte Carlo simulation for cone diameters of 4, 7.5, 10, and 15 mm. As developed by Das et al.,^24^ Tyler et al.,^25^ Yarahmadi et al.^26^ and many other authors, the ionization chamber size, even the pinpoint type (Fig. 3), is too big and shows the widest penumbra for all the cones. The excellent sensitive volume of the MicroDiamond (0.004 mm^3^) is thwarted by the big diameter of the detector: 2.2 mm. This explains the small difference obtained with MC on the penumbra (+0.45 mm for cone diameter at 4 mm), as shown in Fig. 4.

**Figure 5:**
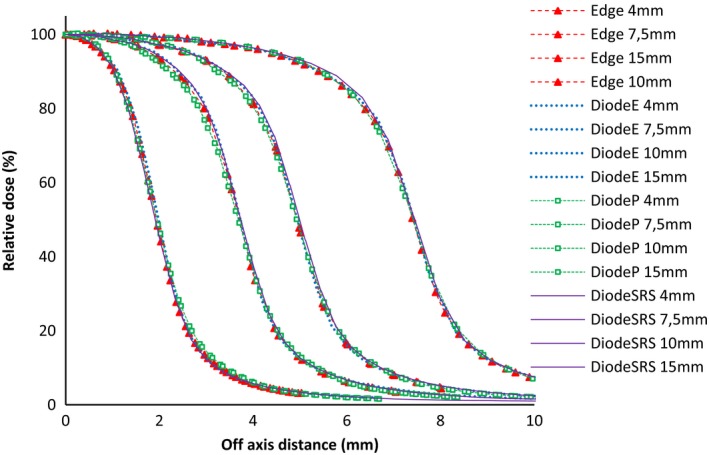
Beam profiles comparison for all the diode detectors (E, P, Edge, and SRS) for cone diameters of 4, 7.5, 10, and 15 mm.

Figure 5 represents the beam profiles for all the diode based detectors (E, P, Edge, and SRS) for cone diameters of 4, 7.5, 10, and 15 mm. One can observe a perfect superimposition of all the curves (maximum difference of ±0.1 mm), which suggests that they all respond within the same way.

**Figure 6:**
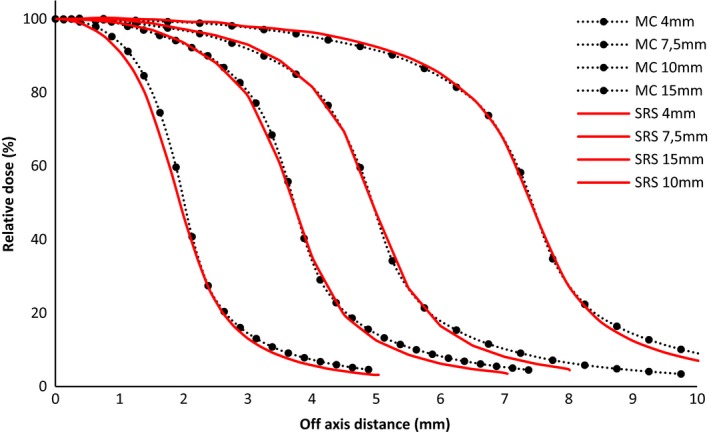
Beam profiles comparison between diode SRS and Monte Carlo simulation for cone diameters of 4, 7.5, 10, and 15 mm.

On Fig. 6, we can see (within ±0.2 mm) a good agreement between the diode SRS and the MC simulation of beam profiles for all the cone diameters.

**Figure 7:**
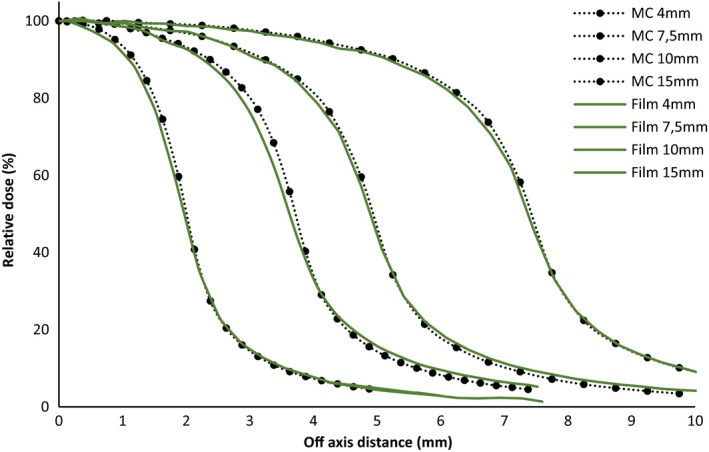
Beam profiles comparison between EBT3 film and Monte Carlo simulation for cone diameters of 4, 7.5, 10, and 15 mm.

EBT3 Gafchromic films are known to have a high spatial resolution^6^, ^25^, ^26^ explaining the proximity of these results with those of MC (Fig. 7). This agreement is within ±0.15 mm for all the curves.

### 3.D PDD results

**Figure 8:**
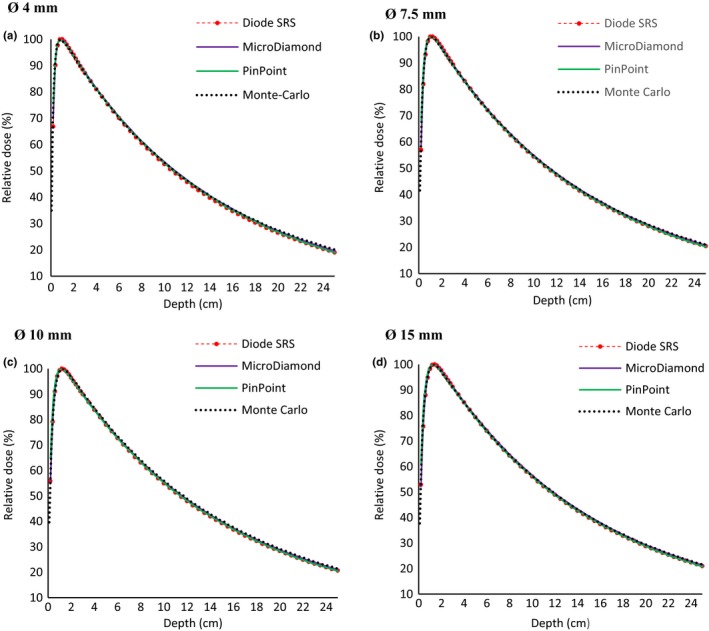
Percent depth‐dose comparison between measurements with diode SRS, MicroDiamond, pinpoint, and Monte Carlo simulation for cone diameters respectively of 4 (a), 7.5 (b), 10 (c), and 15 mm (d).

Figure 8 show the PDD measured with SRS diode, MicroDiamond, pinpoint detectors. and calculated with our reference (MC simulation), for cone diameters of 4, 7.5, 10, and 15 mm. It should be noted that for the depth‐dose measurements no corrections were made. The local percentage deviation with MC from the buildup region for the pinpoint, the SRS, and the MicroDiamond goes up respectively to more than 10%, 5%, and 2% whatever the cone diameter is. In the decreasing part of the PDD curve, the agreement between MC and all the detectors is quite good. The maximum deviations for the pinpoint, the SRS, and the MicroDiamond are respectively of 0.5%, 1%, and 0.5% for the 4‐mm diameter cone, and 0.6%, 0.6%, and 0.3% for the 15 mm one. All these results are in agreement with those reported in the literature.^12^, ^25^, ^27^

## 4 DISCUSSION

**Figure 9:**
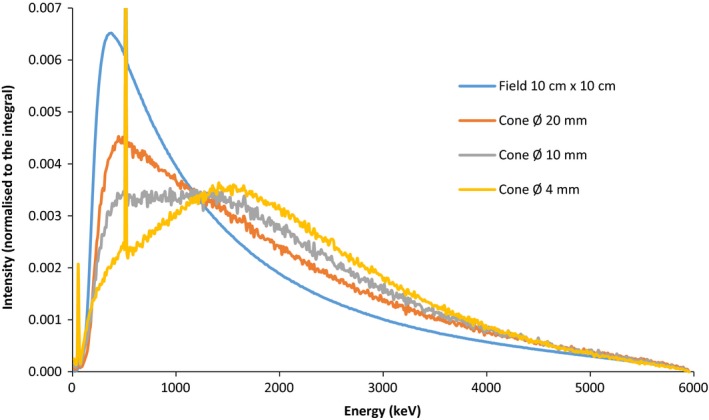
Energy spectrum variation of photon in air on the surface of the water phantom for different field sizes: 10 × 10 cm, cones with diameters of 20, 10, and 4 mm. Spectra are simulated with PenEasy Monte Carlo simulation. The peak represents the electron‐positron annihilation peak at 511 keV. It is presents for all field sizes.

According to report 103 of IPEM,^28^ MV photon beam was defined as “small field” when the field size is not enough to provide charged particle equilibrium at the position of measurement and the collimation device obstructs part of the direct beam source as viewed from the point of measurement. When the lateral electronic equilibrium is not achieved (field size less than the lateral range of secondary electrons), electrons of lowest energy are missing on the beam axis causing an increase of the electronic spectrum average energy. At the same time, decreasing field size causes photonic spectrum modification (Fig. 9) that induce in turn spectrum modification of secondary electrons.

These effects will be directly in relation with the nonwater equivalence detectors (density and composition). Indeed, for these detectors, the electron stopping power ratios^24^, ^29^ and the absorption coefficient ratios of photons between water and detector material vary according to the electronic energy spectrum.

**Figure 10:**
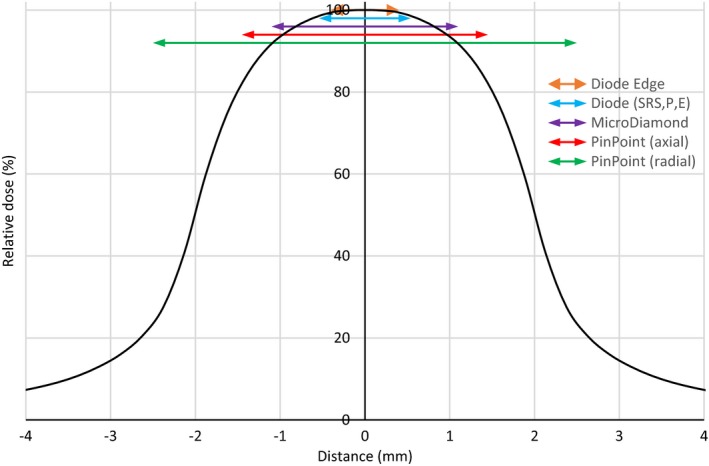
1D Dose profile perpendicular of a 4‐mm diameter cone photon field. Colored arrows indicate the sizes of the different detectors used.

In addition, the size of the detector used for the OF estimation and beam profiles measurements has a crucial importance to limit the partial volume effect. Figure 10 shows a 1D perpendicular dose profile of a 4‐mm diameter cone for a 6‐MV photon beam and the size of the different active detectors: MicroDiamond, pinpoints, diodes SRS, P, E, and Edge.

In Fig. 2, one can classify the detectors according to their response: the diodes and the MicroDiamond overestimate the OF, the ionization chambers underestimate the OF and the film whose the response is equivalent of the MC as reference values. For a cone diameter higher than 12.5 mm, all the studied detectors are able to estimate OF with ±2%. This result is in accordance with Charles et al.^30^ who determines that a field size was “small” when the square is less than 12 mm for a 6‐MV beam. For smaller cones, the size of the detectors is the main reason in the underestimation of OF or the disagreement between measured beam profiles and MC‐simulated ones: it is the case of the pinpoint ionization chamber due to its largest air cavity volume and the induced partial volume effects.^7^, ^22^

Diodes are the smallest detectors and are commonly used for dosimetry because of their high spatial resolution and small sensitive volume. They are good candidates for PDD and off‐axis measurements. However, the overestimation response of these detectors for OF determinations results from the energy, angular, dose rate dependences, and the high density of silicon (Table 1) in comparison with water.^4^, ^8^, ^27^, ^31^

For the shielded diodes (Edge and P) the deviation with the MC simulations on the OF measurements is greater than that obtained for the unshielded ones (SRS and E). The larger gap is attributed to increased perturbation of the local particle fluence caused by the presence of tungsten or copper high atomic number materials used as backing medium in the diode P (PTW 60008) and the diode Edge respectively.^11^ Finally, one can see that the major studies using diodes for SRS treatment with circular cone definitely introducing correction factors for OF determination.^4^, ^8^, ^31^, ^32^

The MicroDiamond detector which is a synthetic diamond material overestimates the OF with a maximum gap of 3.7%. This detector has a good signal‐to‐noise ratio and a much better water equivalence than the other studied diode detectors, its size (Fig. 9) is small with active sensitive volume of 0,004 mm^3^. Morales et al.^27^ show that in a 6‐MV SRS Novalis Trilogy linear accelerator equipped with BrainLAB circular cones (30–4 mm diameters), MicroDiamond detector possesses good dosimetric properties. Chalkley et al.^31^ made the same study on a Cyberknife system and concluded that the MicroDiamond is an excellent detector. These findings are in agreement with the present work for OF, PDD, and beam profile determination.

The very good results of EBT3 film on OF and beam profiles measurements, confirmed in many publications,^6^, ^8^, ^23^ is close to being a perfect detector: dosimetrically water equivalent, high spatial resolution, and minimal energy dependence.^33^ However, it is complicated to use, not a real‐time dosimeter and can have some uncertainties due to film polarization, scanner non‐uniformity and handling techniques.^25^ However, for the first OF determination of a new machine (Cyberknife, linear accelerator), radiochromic film is an unavoidable detector with recognized accuracy.

## 5 CONCLUSION

Monte Carlo simulation, as our gold standard, helps us to determine, over the wide range of detectors we used the most appropriate for measuring the OF, beam profiles and PDD. It is confirmed here that the radiochromic film, especially EBT3 film is the more accurate detector for OF and off axis profile determination of stereotactic cones but it is restrictive to use. Due to inappropriate size of sensitive volume and composition of respectively the pinpoints and the diodes, these detectors do not seem to be suitable without OF corrective factors particularly for cones with diameters below 10 mm. Nevertheless, these diodes are effective and recommended for beam profiles and PDD measurements whatever the cone diameter is. Finally, despite its sensitive volume size MicroDiamond seems to be a good consensual detector for OF determination for all used cones.

## CONFLICT OF INTEREST

The authors declare that they have no conflict of interest.
